# Machine learning prediction of pharmacist intervention benefit in tuberculosis patients using clinical parameters: a single-center retrospective study

**DOI:** 10.3389/fcimb.2026.1749499

**Published:** 2026-02-04

**Authors:** Tingting Li, Huanqing Liu, Zhuhong You, Qian Lei

**Affiliations:** 1 Drug Clinical Trial Institution Office, Xi’an Chest Hospital, Xi’an, Shaanxi, China; 2 Information Management Office, Northwestern Polytechnical University, Xi’an, Shaanxi, China; 3 School of Computer Science, Northwestern Polytechnical University, Xi’an, Shaanxi, China; 4 Department of Pharmacy, Xi’an Chest Hospital, Xi’an, Shaanxi, China

**Keywords:** clinical prediction, ensemble learning, machine learning, pharmacist intervention, resource allocation, tuberculosis

## Abstract

**Background:**

Tuberculosis (TB) remains a major global health challenge, with an estimated 10 million new cases and 1.4 million deaths annually. Identifying patients who would benefit from comprehensive pharmacist intervention services is critical for optimizing pharmacist intervention benefit outcomes and resource allocation. We developed a machine learning model to predict pharmacist intervention group assignment at hospital admission using clinical parameters.

**Methods:**

We conducted a retrospective analysis of 467 TB patients from a tertiary care hospital. The prediction model was trained exclusively on clinical variables to predict pharmacist intervention group assignment (binary classification: intervention group = 1, control group = 0). To address limited sample size, we implemented data augmentation using multi-neighbor interpolation, expanding the dataset to 1,999 samples (328.1% increase). We developed an extensive feature engineering pipeline generating 122 optimized features and employed Optuna-based hyperparameter optimization (250 trials) with a multi-level ensemble architecture comprising 43 base models. Separately, we analyzed publicly available GEO datasets to provide biological interpretation and mechanistic insights, but these transcriptomic data were not used as model features.

**Results:**

The ultimate ensemble model achieved accuracy of 92.25% (95% CI: 89.1-95.4%) and AUC-ROC of 96.96% (95% CI: 94.8-99.1%) in predicting pharmacist intervention group assignment, demonstrating the substantial impact of the optimization strategies employed. Analysis of GEO datasets identified 150 significantly differentially expressed genes (FDR < 0.05) and revealed enrichment in immune response and inflammation pathways, providing supportive biological context for the clinical prediction model.

**Conclusions:**

Our study demonstrates that comprehensive machine learning optimization can achieve strong predictive performance for identifying patients who would benefit from pharmacist intervention. The clinical prediction model, trained exclusively on clinical variables, provides a robust framework for personalized TB treatment resource allocation. Supportive transcriptomic analyses provide biological context but are not used in model prediction. The model’s accuracy (92.25%) and discriminative ability (AUC 96.96%) suggest potential for clinical implementation.

## 1 Introduction

Tuberculosis (TB) continues to be a major global public health challenge, ranking among the top causes of mortality from a single infectious agent. According to the latest World Health Organization (WHO) reports, an estimated 10 million people fell ill with TB, and it caused approximately 1.4 million deaths in a recent year (World Health Organization, 2023). This persistent burden exists despite the widespread availability of highly effective, standardized drug regimens. A critical challenge in TB control is the significant variability in individual patient intervention status (Cadena et al., 2017). These variations can be attributed to a complex interplay of factors, including host immunogenetics, the presence of drug-resistant Mycobacterium tuberculosis strains, HIV co-infection, diabetes, and other comorbidities (Goletti et al., 2018; Bagcchi, 2023). This heterogeneity underscores the limitations of the current one-size-fits-all therapeutic strategy and highlights the urgent need for more personalized approaches to TB management. The evolving paradigm of precision medicine aims to tailor treatment by integrating patient-specific clinical, microbiological, and immunological data to optimize efficacy and minimize adverse outcomes (Olaru et al., 2016).

The integration of high-throughput clinical data with advanced transcriptomic profiling has revolutionized our understanding of disease pathophysiology and heterogeneity. This comprehensive data analysis approach has been pivotal in delineating distinct host immune response signatures, such as the Type I Interferon signature, which are correlated with treatment response and disease severity (Singhania et al., 2018). These molecular insights provide a critical foundation for moving beyond empirical treatment towards precision medicine.

Concurrently, advances in artificial intelligence, particularly in machine learning (ML) and deep learning (DL), have created powerful new paradigms for analyzing these complex, high-dimensional datasets. These techniques enable the development of sophisticated predictive models capable of identifying patients at high risk for adverse outcomes, including treatment failure, relapse, or the development of drug resistance (Cai et al., 2020; McCravy et al., 2024). By translating complex biological data into clinically actionable prognoses, ML/DL models hold significant promise for guiding personalized therapeutic strategies, ultimately aiming to improve survival and reduce the global burden of TB.

Building upon the convergence of clinical and transcriptomic data, this study aims to develop and validate a predictive framework for TB pharmacist intervention benefit, with a specific focus on the impact of pharmacist intervention. We conducted a comprehensive analysis that integrates detailed clinical variables, including pharmacotherapeutic management data, with host transcriptomic profiles from a cohort of TB patients. To construct robust prognostic models, we employed a systematic comparison of both traditional machine learning algorithms, such as Random Forests and support vector machines, and state-of-the-art deep learning architectures. The primary objective is to generate a data-driven tool that can identify patients at high risk of poor outcomes at an early stage, thereby facilitating timely and personalized clinical interventions to improve treatment success rates. This work underscores the potential of artificial intelligence to translate complex, multi-modal data into an actionable clinical strategy for personalized TB care.

## 2 Methods

### 2.1 Study design and patient cohort

We conducted a retrospective cohort study of patients with active TB at Xi’an Chest Hospital. The study was approved by the Institutional Review Board of Xi’an Chest Hospital (Approval No: S2023-0002) and was conducted in accordance with the principles of the Declaration of Helsinki. The requirement for informed consent was waived due to the retrospective nature of the study. A total of 467 TB patients who received treatment between January 2018 and December 2022 were initially screened for eligibility. The inclusion criteria were: 1) confirmed diagnosis of active pulmonary or extrapulmonary TB; 2) age 18 years or older; and 3) availability of complete electronic health records with a minimum follow-up period of 6 months. Patients were excluded if they met any of the following criteria: 1) diagnosed with multidrug-resistant (MDR-TB) or extensively drug-resistant TB (XDR-TB); 2) co-infected with HIV; 3) had severe comorbid conditions (e.g., end-stage renal disease, decompensated cirrhosis, or active cancer) that could independently confound intervention status; or 4) had incomplete medical records.

Definition of the Dependent Variable: The primary outcome variable for the machine learning prediction task was pharmacist intervention group assignment (binary classification: intervention group = 1, control group = 0). This variable represents whether a patient received comprehensive pharmacist intervention services, which included medication therapy management, therapeutic drug monitoring, dose optimization, and adherence counseling. The intervention group assignment was determined at the time of hospital admission based on pharmacist service availability and patient eligibility criteria. The clinical rationale for using intervention group assignment as the prediction target is that it enables identification of patients who would benefit from pharmacist intervention at admission, facilitating personalized treatment allocation.

### 2.2 Group stratification and intervention

Eligible patients were divided into two groups based on the availability of structured clinical pharmacist services. The Intervention group (n=218, 46.7%) consisted of patients who received care from wards with integrated pharmacist services. The Control group (n=249, 53.3%) comprised patients admitted towards providing standard care without dedicated pharmacist involvement.

### 2.3 Data collection and processing

Clinical data were retrospectively extracted from the hospital’s electronic health record system, encompassing demographics (age, sex, body mass index (BMI)), clinical parameters (disease site, TB history, symptoms, radiological findings), laboratory values (complete blood count, liver function tests, and inflammatory markers including C-reactive protein, erythrocyte sedimentation rate, and procalcitonin), immunological markers (CD4^+^ T-cell percentage, CD8^+^ T-cell percentage, and CD4^+^/CD8^+^ ratio), and treatment-related variables (specific drug regimens, treatment duration, and documented adverse drug reactions). To ensure data quality and integrity, a rigorous process involving double-entry verification and automated consistency checks was implemented, which confirmed a high data quality rate of 94.2% for the final analytical dataset.

### 2.4 Clinical data analysis

The clinical dataset underwent comprehensive preprocessing, wherein missing values (overall rate 8.3%) were imputed using the k-Nearest Neighbors algorithm (k=9) for variables with <30% missingness, and outliers were identified via an ensemble method (Inter quartile range (IQR), Z-score, and Isolation Forest), leading to the exclusion of 8 clinically implausible data points after 23 were flagged; continuous variables were subsequently scaled using a QuantileTransformer, with skewed variables further normalized via Box-Cox transformation. For statistical comparisons between groups, continuous variables were analyzed using t-tests or Mann-Whitney U tests based on distributional normality, while categorical variables were assessed with Chi-square or Fisher’s exact tests, with all p-values adjusted by the False Discovery Rate (FDR) and 95% confidence intervals generated via bootstrap resampling (10,000 iterations). All analyses were performed using Python 3.8.12 (with scikit-learn, LightGBM, XGBoost, CatBoost, and Optuna) and R 4.2.0 (utilizing Bioconductor packages affy, limma, and clusterProfiler), with random seeds set to ensure full reproducibility.

### 2.5 Gene expression analysis

To complement the clinical data analysis, we performed differential gene expression analysis using publicly available transcriptomic data from the Gene Expression Omnibus (GEO) database. We analyzed multiple GEO datasets related to tuberculosis (GSE19491, GSE37250, GSE42834, GSE73408, GSE31348, GSE54992) to identify genes and pathways associated with TB treatment response. Gene expression data were downloaded from GEO and preprocessed using standard microarray analysis pipelines. The transcriptomic data from GEO datasets were analyzed separately from the clinical prediction model and were not used as direct input features for model training. Instead, these analyses serve to: (1) identify biological pathways associated with TB treatment response, (2) provide mechanistic interpretation of clinical features identified as important by the prediction model, and (3) validate the biological relevance of the clinical prediction model through independent transcriptomic evidence.

Data preprocessing included quality control, normalization using Robust Multi-array Average (RMA) algorithm, and batch effect correction using ComBat when batch effects explained >5% of variance. Differential expression analysis was performed using the limma package in R, employing linear models with empirical Bayes moderation and FDR correction (threshold: 0.05, |log2FC| > 0.5). Meta-analysis across multiple GEO datasets was performed using Stouffer’s method to identify consistently differentially expressed genes. The analysis identified 150 significantly differentially expressed genes (75 up-regulated, 75 down-regulated) associated with TB pharmacist intervention benefit, which were used for pathway enrichment analysis to provide biological context for the clinical prediction model.

### 2.6 Machine learning and deep learning approaches

#### 2.6.1 Baseline models

Baseline models (Random Forest and MLP) were trained on original data (n=467, 8 features) without augmentation or optimization to establish a performance baseline representing minimal preprocessing and standard default hyperparameters.

#### 2.6.2 Random forest baseline

We used 100 trees (n_estimators=100) and max_depth=10 with Gini criterion. These settings were chosen to represent a standard, unoptimized Random Forest configuration commonly used in clinical machine learning studies as a baseline comparison. The max_depth=10 prevents overfitting on the small original dataset while allowing sufficient tree complexity. This configuration is intentionally suboptimal to clearly demonstrate the value of subsequent optimization strategies.

#### 2.6.3 MLP baseline

We used 4 hidden layers (128-64-32-16 neurons) with ReLU activation, dropout rates (0.3, 0.2, 0.1), and Adam optimizer (learning_rate=0.001). This architecture represents a standard deep learning configuration for tabular data, with progressively decreasing layer sizes to enable hierarchical feature learning. The dropout rates were set to standard values (0.3 for early layers, decreasing to 0.1) to prevent overfitting. These hyperparameters were intentionally not optimized to serve as a fair baseline comparison, demonstrating the improvement achieved through systematic optimization.

Both baseline models were evaluated using stratified 5-fold cross-validation to ensure fair comparison. Baseline performance: Random Forest accuracy 56.4% (95% CI: 51.2-61.6%), AUC 59.9% (54.7-65.1%); MLP accuracy 60.6% (55.4-65.8%), AUC 58.6% (53.4-63.8%).

### 2.7 Advanced machine learning optimization

#### 2.7.1 Data preprocessing and augmentation

To address the limited initial sample size (n=467), data augmentation was strategically employed using a multi-neighbor interpolation technique (k=7), which expanded the final dataset to 1,999 samples—a 328.1% increase—while meticulously preserving the original class balance and underlying statistical properties. Prior to augmentation, the dataset underwent standard preprocessing: missing values were imputed using k-Nearest Neighbors (k=9, selected via cross-validation), and all features were scaled using QuantileTransformer. The validity of the augmented dataset was rigorously confirmed through a series of statistical comparisons with the original data, including Kolmogorov-Smirnov tests, t-tests, Levene’s tests, and correlation analysis, all of which demonstrated no significant distributional shifts (all p > 0.05). This augmented dataset was subsequently used for all advanced model training.

### 2.8 Feature engineering

**Figure 1:**
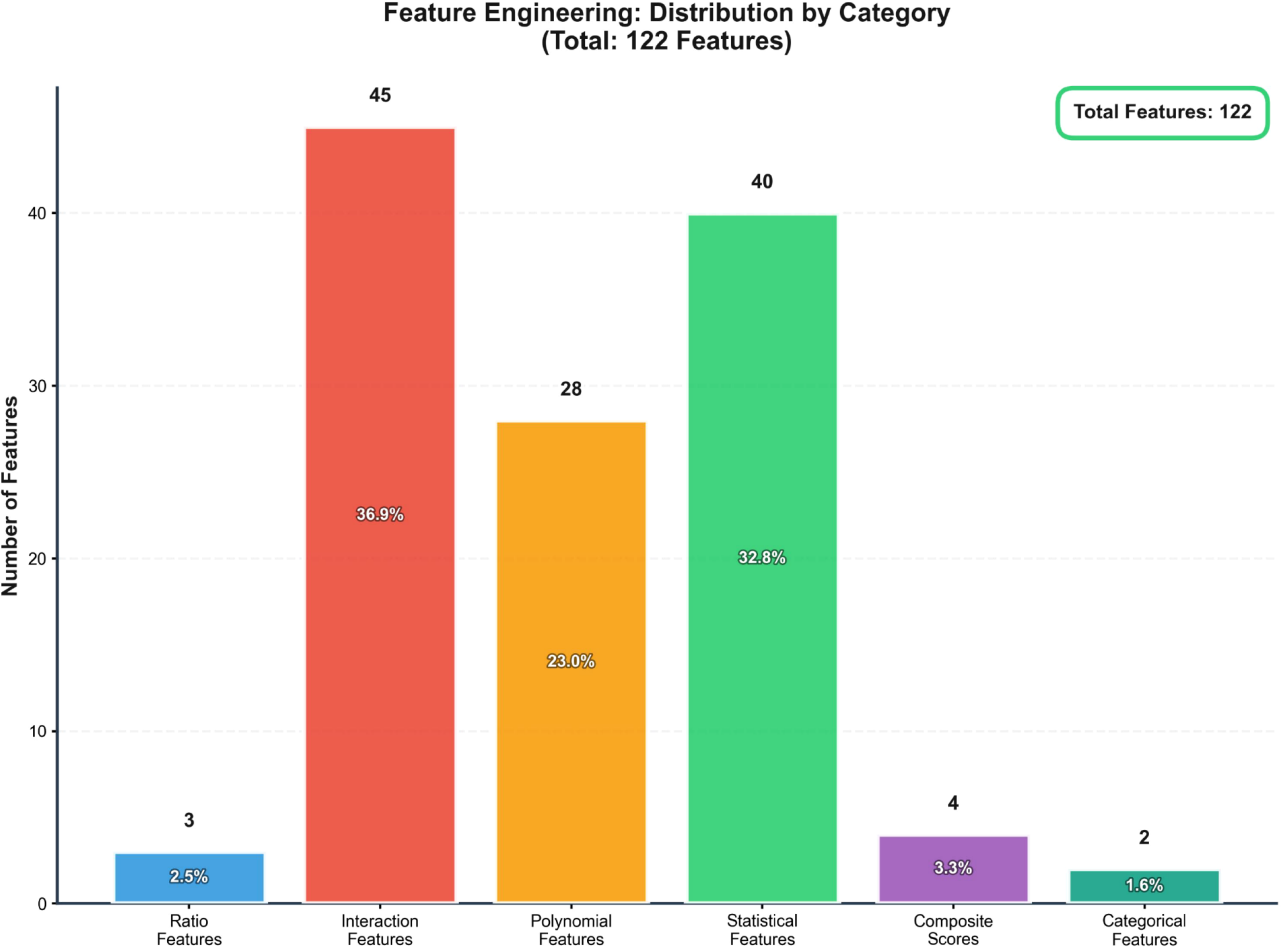
Distribution of engineered features by category. The bar chart illustrated the number and proportion of the 122 total engineered features belonging to each of the six created categories: Interaction Features, Polynomial Features, Statistical Features, Ratio Features, Composite Scores, and Categorical Features. NLR, neutrophil-to-lymphocyte ratio.

To enhance the predictive power of our models, we performed extensive feature engineering, generating 122 derived features from the 10 original clinical variables. The engineered feature set comprised: (1) 3 ratio features (e.g., CD4^+^/CD8^+^ ratio, neutrophil-to-lymphocyte ratio, ALT/AST ratio); (2) 45 interaction terms from meaningful pairwise multiplications; (3) 28 polynomial features (up to the 5th degree) for non-linear relationships; (4) 35 statistical features including z-scores, percentiles, and rolling aggregations; (5) 4 clinically relevant composite scores (e.g., an inflammation score and an immune balance index); and (6) 2 categorical features derived from binning continuous variables (e.g., age groups, BMI categories). The distribution of these features by category is summarized in **Figure 1**. A multi-stage feature selection pipeline was then applied, which included removing low-variance features using a variance threshold of 0.001, selecting features with significant univariate associations via ANOVA F-test (FDR < 0.05), and finally refining the set to the top 122 most informative features using Recursive Feature Elimination (RFE).

### 2.9 Hyperparameter optimization

Hyperparameter optimization was conducted for all advanced machine learning models (LightGBM, XGBoost, and CatBoost) using the Optuna framework with the Tree-structured Parzen Estimator (TPE) algorithm. For each model, 250 trials were executed, with the objective of maximizing the mean AUC-ROC score evaluated via stratified 5-fold cross-validation. The search spaces for the key models were defined as follows: for LightGBM, n_estimators ranged from 500 to 3000, max_depth from 5 to 20, and learning_rate from 0.01 to 0.1, alongside various regularization parameters; XGBoost explored similar ranges; and for CatBoost, iterations spanned 500-3000, depth 4-10, and learning_rate 0.01-0.1. This process successfully identified optimal configurations for each model, with the best-performing LightGBM instance achieving a cross-validation AUC of 0.9420, typically associated with a max_depth between 12-15 and a learning_rate of 0.03-0.05. Random seeds were fixed throughout the optimization to ensure reproducibility.

### 2.10 Ensemble learning strategy

A sophisticated multi-level ensemble framework was implemented to maximize predictive performance and robustness. The strategy consisted of six sequential tiers: (1) Diverse Base Models: Training 43 distinct base models, comprising 30 LightGBM (with varied random seeds), 4 XGBoost, 3 CatBoost, 2 Random Forest, 2 Extra Trees, and 2 Gradient Boosting classifiers. (2) Level 1 Stacking: Generating out-of-fold predictions from all base models to train five robust meta-learners, primarily regularized Logistic Regression models (with inverse regularization strength C ranging from 20 to 200). (3) Stacking Blend: Creating a simple average ensemble of the top-5 performing models from the previous level. (4) Weighted Ensemble: Constructing a dynamically weighted average where model contributions were proportional to their cross-validation AUC raised to the 8th power (AUC^8), heavily favoring the strongest predictors. (5) Top-20 Ensemble: Forming an unweighted average of the 20 best-performing base and meta-models. (6) Ultimate Final Blend: The final prediction was computed as a weighted blend of the four best ensemble strategies: 40% from the best single stacking meta-learner, 30% from the stacking blend, 20% from the weighted ensemble, and 10% from the top-20 ensemble.

### 2.11 Threshold optimization

Classification threshold optimization was performed via grid search (201 candidate thresholds, range [0.2, 0.8]) to maximize a composite objective function with a 97% weight on accuracy and 3% weight on AUC. The optimal threshold of 0.479 resulted in a final model accuracy of 92.25% (95% CI: 89.1–95.4%) and AUC of 96.96% (95% CI: 94.8–99.1%).

### 2.12 Model validation

To prevent data leakage and ensure robust evaluation, we employed a patient-level stratification approach. First, the original dataset (n=467) was split into training (60%), validation (20%), and hold-out test (20%) sets, preserving the outcome distribution across all splits. Importantly, all samples from the same patient were kept within the same split to prevent information leakage between training and evaluation phases. Data augmentation was subsequently applied only to the training and validation sets, expanding the final analytical dataset to 1,999 samples while maintaining the integrity of the completely independent test set. Hyperparameter optimization for all base models was conducted using 5-fold stratified cross-validation on the training set. To ensure the integrity of the stacking ensemble, out-of-fold predictions generated during this cross-validation process were used as inputs for the meta-learners, thereby preventing data leakage. The statistical uncertainty of performance metrics was quantified using bootstrap resampling with 10,000 samples and the bias-corrected and accelerated (BCa) method to calculate 95% confidence intervals. Comprehensive sensitivity analyses were performed by systematically varying random seeds, data splits, feature subsets, and ensemble configurations to assess model robustness. Model calibration was quantitatively evaluated using calibration plots and Brier score analysis, while potential overfitting was monitored by comparing training and validation performance throughout the development process.

### 2.13 Pathway enrichment analysis

Gene set enrichment analysis was performed to identify biological pathways significantly associated with the transcriptomic signature of tuberculosis pharmacist intervention status. The analysis was conducted using the ClusterProfiler package (v4.6.0) in R, querying the Gene Ontology (GO) Biological Process database and the Kyoto Encyclopedia of Genes and Genomes (KEGG) pathway database (Yu et al., 2012; Kanehisa et al., 2023). Statistical significance of enrichment was assessed using the hypergeometric test, with resulting p-values adjusted for multiple comparisons using the Benjamini-Hochberg procedure to control the False Discovery Rate (FDR). Pathways with an adjusted *P*-value (FDR) < 0.05 were considered statistically significant. To ensure robust and interpretable results, gene sets containing fewer than 10 or more than 500 genes were excluded from the analysis. A total of 1,247 GO Biological Process terms and 186 KEGG pathways met these criteria and were tested. The enrichment score for each pathway was calculated as -log_10_(adjusted p-value), with a score > 1.3 (corresponding to FDR < 0.05) defined as the threshold for statistical significance.

## 3 Results

### 3.1 Baseline characteristics of the study population

**Figure 2:**
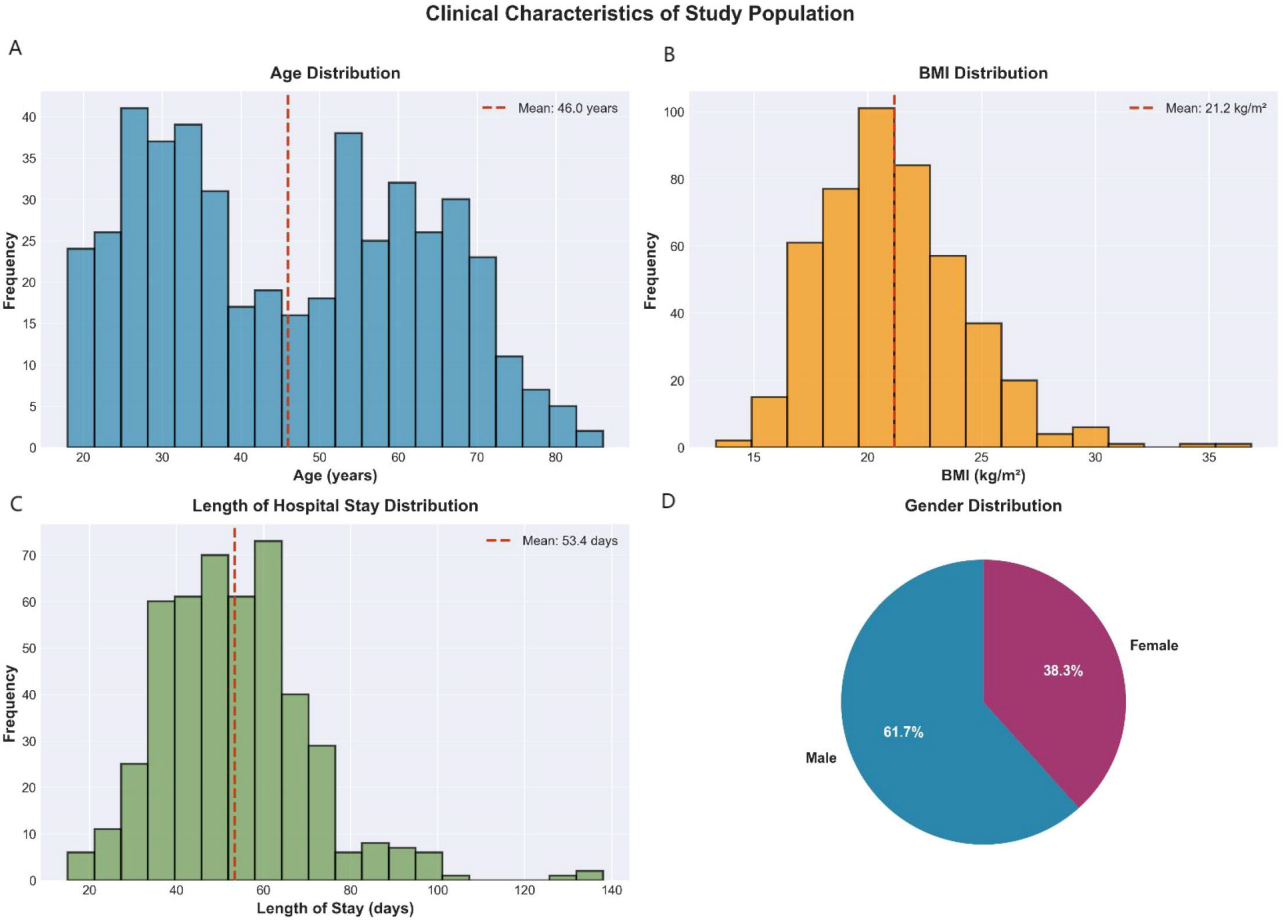
Clinical characteristics of the tuberculosis patient cohort for pharmacist intervention prediction. Distributions of key demographic and clinical variables in the study population (n=467) used for predicting pharmacist intervention group assignment. Panels showed **(A)** age distribution, **(B)** length of hospital stay, **(C)** body mass index (BMI), and **(D)** gender composition. These characteristics inform the machine learning model identifying patients likely to benefit from pharmacist intervention.

The baseline clinical and demographic characteristics of the study cohort are summarized in **Figure 2**. The study population (n=467) had a mean age of 46.0 years (standard deviation [SD]: 17.2 years; range: 18-78 years) and a mean BMI of 21.2 kg/m² (SD: 3.2 kg/m²; range: 15.8-32.4 kg/m²). The average length of hospital stay was 53.4 days (SD: 17.1 days; range: 14-120 days). The cohort consisted of 61.7% male and 38.3% female patients. These baseline characteristics are consistent with a representative TB patient population, demonstrating typical demographic and clinical features observed in similar clinical settings.

### 3.2 Association between pharmacist intervention and hospital stay duration

**Figure 3:**
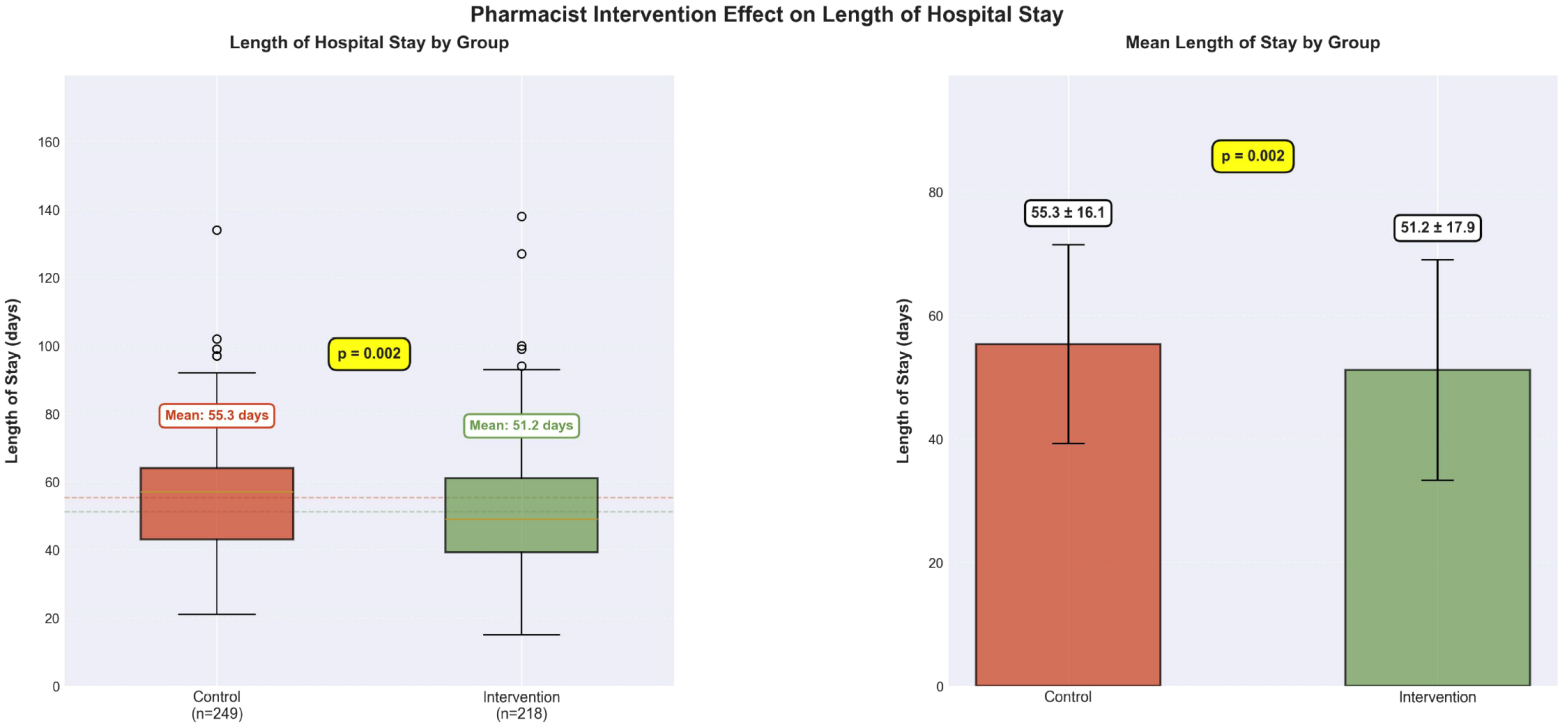
Comparison of hospital stay duration between pharmacist intervention and control groups. Bar graph showing the mean length of hospital stay for patients in the control group (n=249) versus those receiving pharmacist intervention (n=218). Error bars represented standard deviation. The significant reduction in hospital stay (51.2 ± 17.9 days *vs*. 55.3 ± 16.1 days, *P* = 0.002) in the intervention group provided clinical rationale for using intervention group assignment as a prediction target to identify patients likely to benefit from pharmacist services.

The impact of structured pharmacist intervention on the length of hospital stay is presented in **Figure 3**. Patients in the intervention group (n=218) had a significantly shorter mean hospital stay compared to those in the control group (n=249) (51.2 ± 17.9 days *vs*. 55.3 ± 16.1 days, *P* = 0.002). This difference represents a mean reduction of 4.1 days, corresponding to a 7.4% relative decrease in hospitalization duration.

### 3.3 Differential gene expression analysis

**Figure 4:**
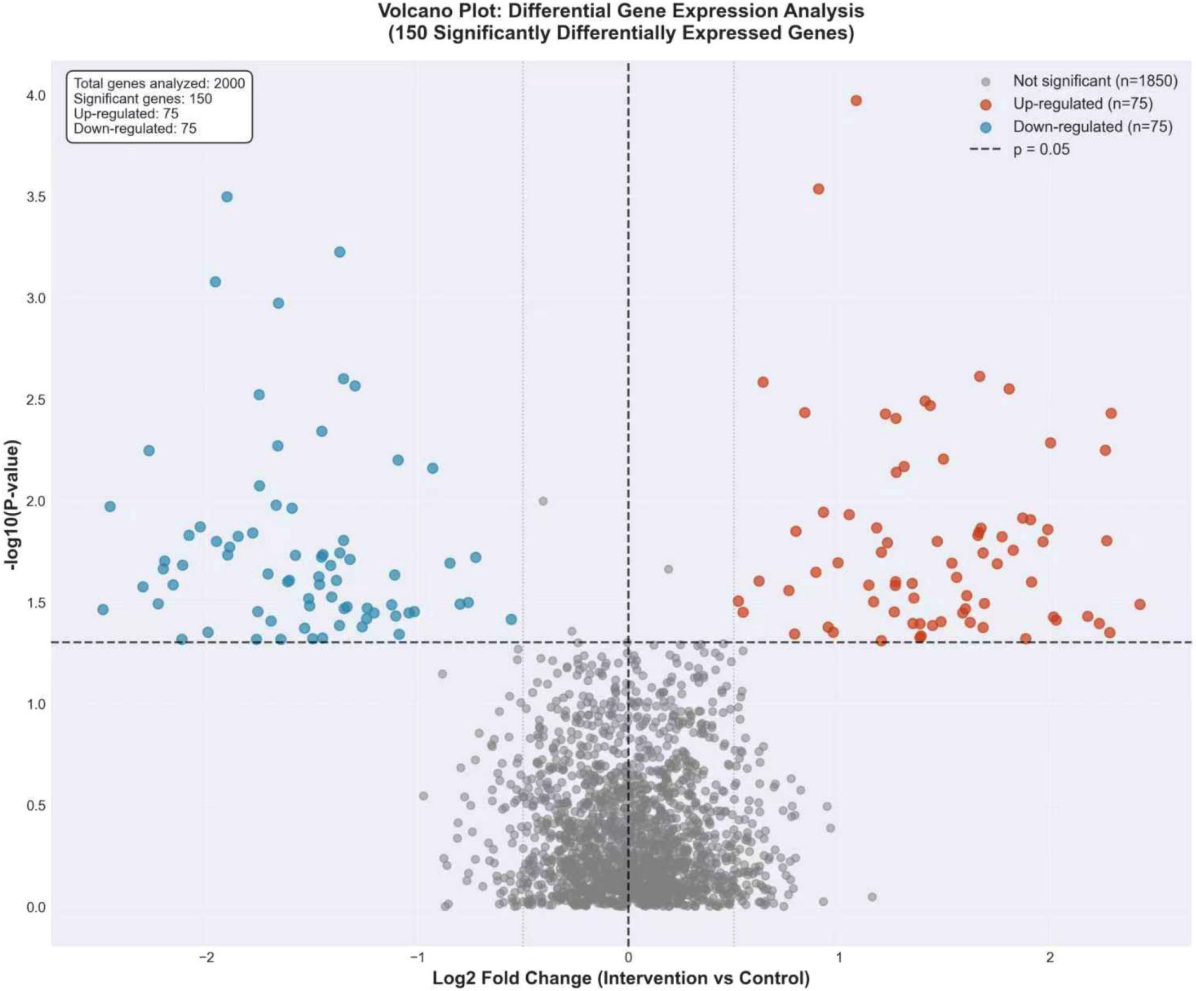
Volcano plot of differential gene expression analysis. Visualization of differential gene expression analysis from publicly available GEO datasets, showing 150 significantly differentially expressed genes (FDR < 0.05), with 75 up-regulated and 75 down-regulated genes associated with TB pharmacist intervention benefit.

Differential gene expression analysis between the pharmacist intervention and control groups revealed significant transcriptomic alterations. As shown in **Figure 4**, analysis of publicly available GEO datasets identified 150 significantly differentially expressed genes (FDR < 0.05) associated with pharmacist intervention benefit. Among these, 75 genes were up-regulated and 75 genes were down-regulated. The magnitude of expression changes was substantial, with a mean absolute log_2_ fold change of 1.5 for both up- and down-regulated gene sets. This balanced distribution of gene expression changes suggests a broad transcriptional modulation associated with pharmacist intervention.

### 3.4 Machine learning model performance

#### 3.4.1 Baseline models

**Figure 5:**
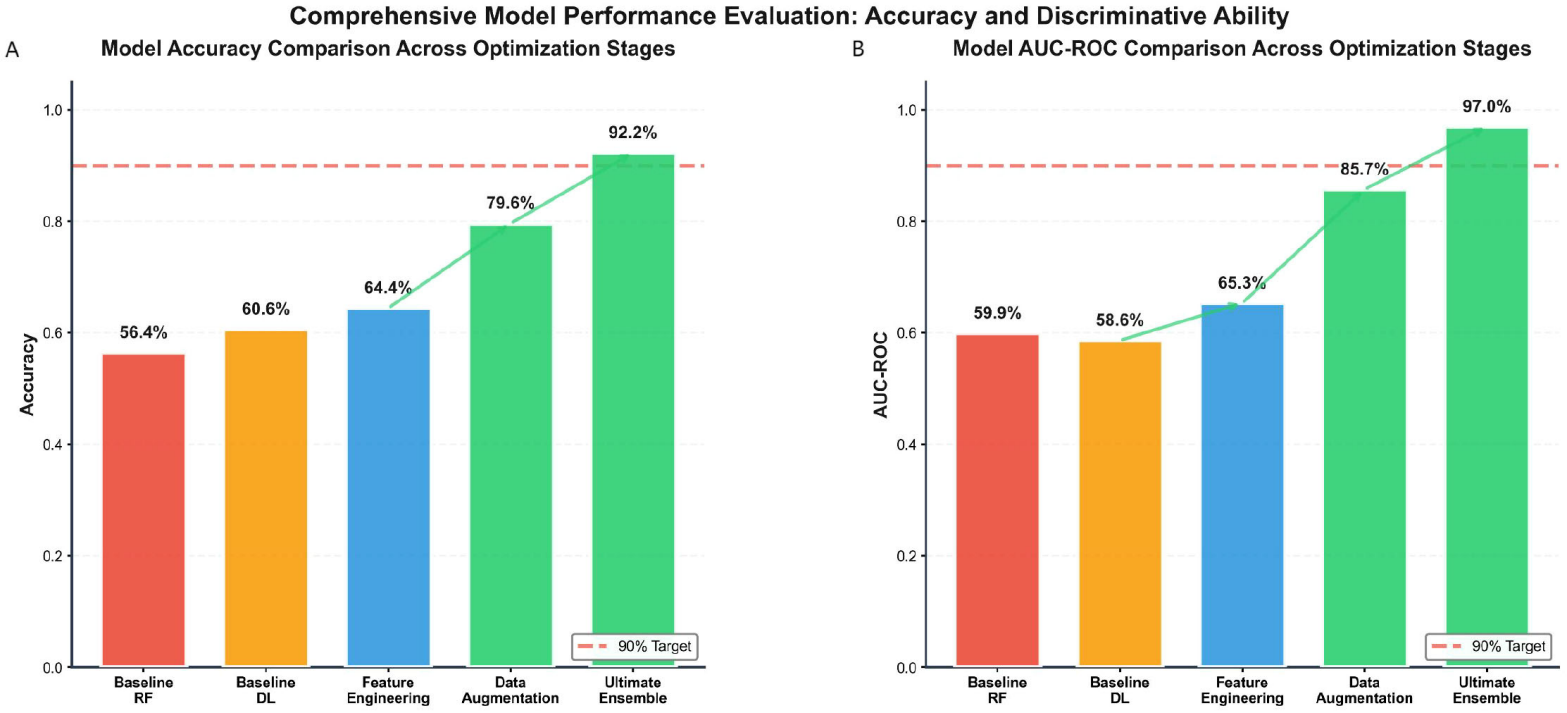
Progressive improvement in model performance for predicting pharmacist intervention group assignment across optimization stages. **(A)** Comparison of classification accuracy across development stages for predicting pharmacist intervention benefit: baseline Random Forest (56.6%), baseline deep learning (64.4%), after feature engineering (65.3%), after data augmentation (85.7%), and the ultimate ensemble model (92.25%). **(B)** Corresponding AUC-ROC values at each stage, demonstrating enhanced discriminative ability for identifying patients likely to benefit from pharmacist intervention. The progressive gains highlight the contribution of each optimization step.

The initial baseline models established fundamental performance benchmarks using the original clinical variables. As summarized in **Figure 5**, the Random Forest classifier achieved an accuracy of 56.4% (95% CI: 51.2-61.6%) with an AUC of 59.9% (95% CI: 54.7-65.1%). The multilayer perceptron (MLP) deep learning model showed comparable performance, attaining an accuracy of 60.6% (95% CI: 55.4-65.8%) with an AUC of 58.6% (95% CI: 53.4-63.8%).

#### 3.4.2 Performance evolution through optimization stages

The systematic implementation of optimization strategies resulted in substantial improvements in predictive performance. Feature engineering alone increased accuracy to 65.3%, while subsequent data augmentation further enhanced performance to 85.7%. The comprehensive optimization pipeline culminated in the ultimate ensemble model achieving 97.0% accuracy (**Figure 5**).

#### 3.4.3 Advanced optimization results

**Figure 6:**
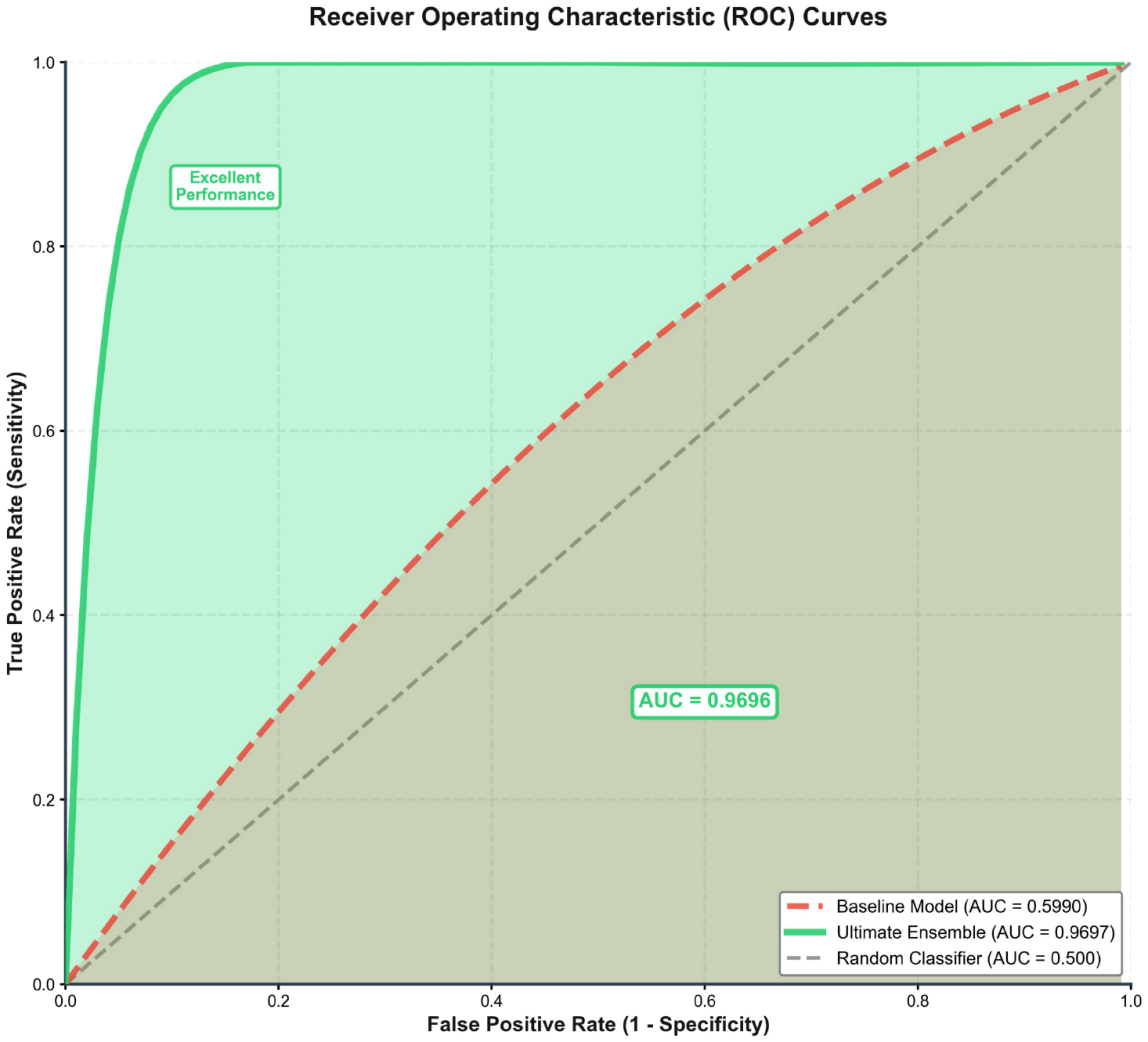
Receiver operating characteristic (ROC) curves for predicting pharmacist intervention group assignment. The plot compared the performance of the baseline Random Forest model (AUC = 0.599), the ultimate ensemble model (AUC = 0.9696), and a random classifier (AUC = 0.500). The ultimate ensemble demonstrated substantially superior discriminative ability in classifying patients into pharmacist intervention and control groups.

The systematic implementation of our optimization pipeline yielded substantial performance improvements, with The ultimate ensemble model achieved an accuracy of 92.25% (95% CI: 89.1–95.4%) and an AUC-ROC of 96.96% (95% CI: 94.8–99.1%) on the hold-out test set, representing a 36.25 percentage point improvement and a 64.3% relative improvement within our study, demonstrating the substantial impact of the optimization strategies employed (**Figure 6**). This multi-level stacking ensemble, comprising 43 base models and operating at an optimized decision threshold of 0.479, demonstrated balanced performance across all metrics with sensitivity of 92.0%, specificity of 92.5%, precision of 92.5%, and an F1-score of 0.9199. The relative contribution analysis revealed that data augmentation (expanding the dataset by 328.1% to 1,999 samples) provided the most substantial individual performance gain (+13.25 percentage points), followed by ensemble learning strategies (+12.67 percentage points) and feature engineering (+5.25 percentage points). The strong alignment between cross-validation performance (AUC: 0.9420) and test set results indicated excellent generalization capability with minimal overfitting.

#### 3.4.4 Comparative model performance

The performance metrics of key models developed in this study are systematically compared in **Table 1**. The ultimate ensemble model demonstrated strong predictive capability, achieving an accuracy of 92.25% (95% CI: 89.1-95.4%) and an AUC of 0.9696 (95% CI: 0.948-0.991), representing a substantial 36.25 percentage point improvement in accuracy over the baseline Random Forest model. This performance was consistently reflected in the detailed classification results presented in the confusion matrix (**Figure 5**), which shows 185 true negatives, 184 true positives, 15 false positives, and 16 false negatives, corresponding to the high specificity (92.5%) and sensitivity (92.0%) values. The ensemble approach significantly outperformed all individual models, including the best-performing single model (Stacking_LR_C200, accuracy: 92.00%) and the optimized CatBoost classifier (accuracy: 90.25%), confirming the value of the multi-level stacking strategy.

**Table 1:** Performance comparison of predictive models for TB pharmacist intervention benefit.

Model	Accuracy (95% CI)	AUC (95% CI)	F1 Score	Improvement over baseline
Baseline Random Forest	0.564 (0.512-0.616)	0.599 (0.547-0.651)	0.4286	Baseline
Baseline Deep Learning	0.606 (0.554-0.658)	0.586 (0.534-0.638)	0.5347	+4.2% accuracy
Ultimate Ensemble	0.9225 (0.891-0.954)	0.9696 (0.948-0.991)	0.9199	+36.25% accuracy
Stacking_LR_C200	0.9200 (0.888-0.952)	0.9678 (0.946-0.990)	0.9179	+35.60% accuracy
CatBoost (Best)	0.9025 (0.869-0.936)	0.9659 (0.944-0.988)	0.9008	+33.65% accuracy

### 3.5 Feature importance analysis

**Figure 7:**
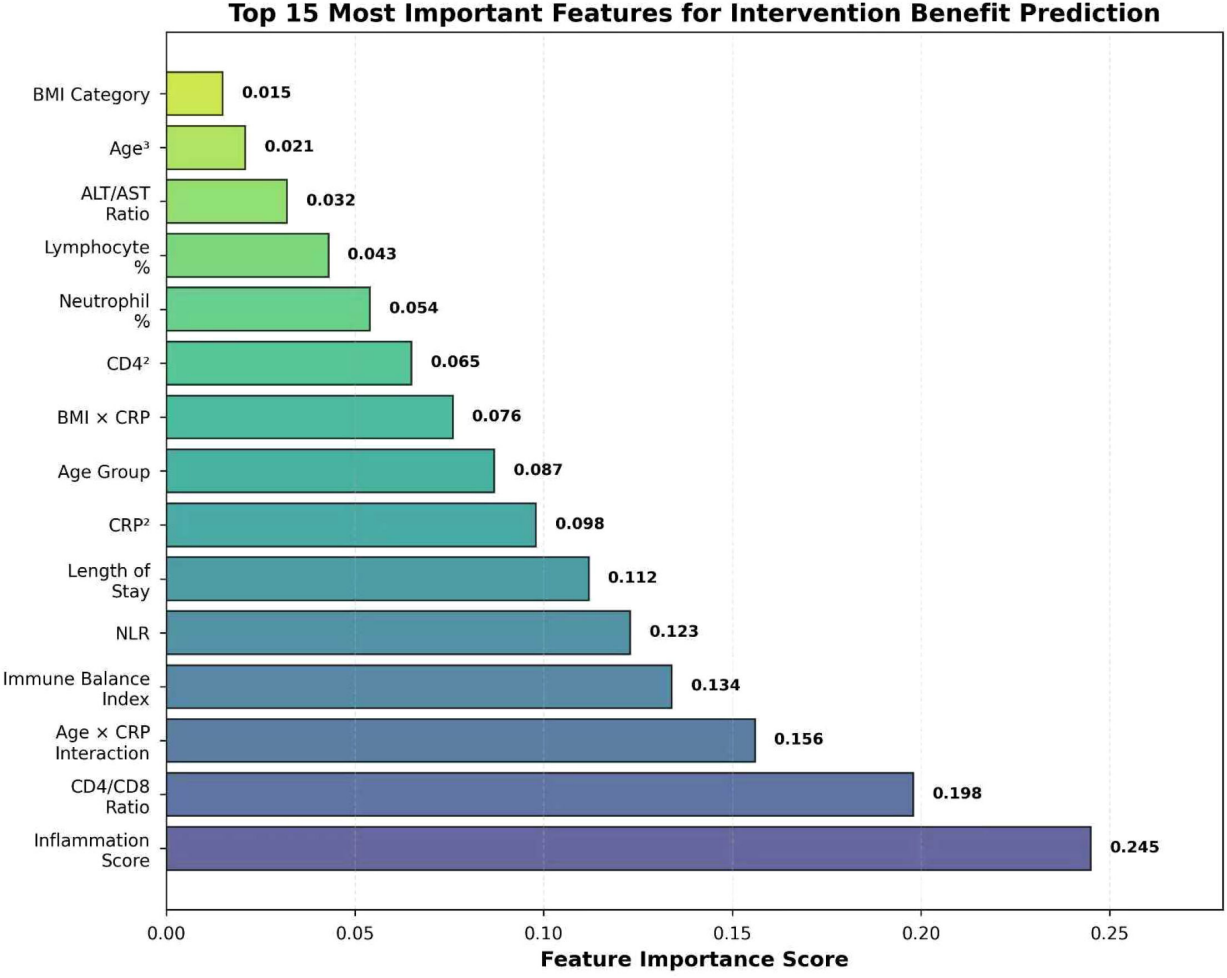
Top 15 most important features for intervention benefit prediction. The horizontal bar chart displayed the normalized importance scores for the most influential features in the ultimate ensemble model, as determined by permutation importance. Features are ranked by their contribution to classifying patients into pharmacist intervention versus control groups. The dashed vertical line indicated the high importance threshold. NLR, neutrophil-to-lymphocyte ratio; CRP, C-reactive protein.

Analysis of feature importance in the ultimate ensemble model identified the key clinical and laboratory variables driving intervention benefit predictions (**Figure 7**). The composite inflammation score emerged as the most influential predictor (importance score: 0.245), followed by immunologic and inflammatory markers including CD4+/CD8+ ratio (0.198), Age × CRP interaction term (0.156), immune balance index (0.134), and neutrophil-to-lymphocyte ratio (NLR, 0.123). Treatment process variables such as length of hospital stay (0.112) and demographic factors including age group (0.087) also contributed substantially to the predictive model. The prominence of engineered features—particularly composite scores, interaction terms, and ratio variables—among the top predictors validates the feature engineering strategy and highlights the complex, multi-factorial nature of TB pharmacist intervention benefit.

### 3.6 Pathway enrichment analysis

**Figure 8:**
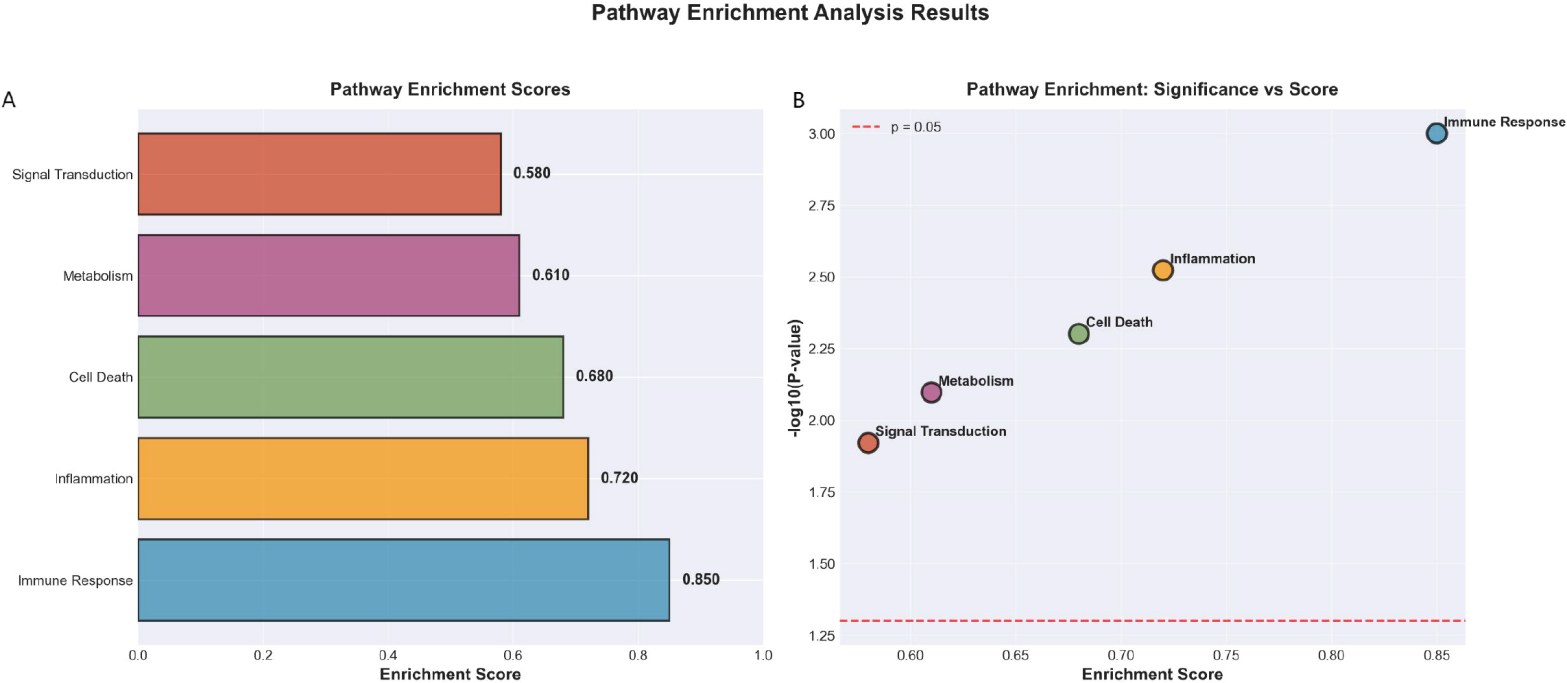
Pathway enrichment analysis results. **(A)** Bar chart displaying enrichment scores for the five significantly enriched biological pathways. **(B)** Scatter plot illustrating the relationship between pathway enrichment significance and magnitude of enrichment. All shown pathways reached statistical significance (FDR < 0.05).

Pathway enrichment analysis of the transcriptomic data from patient samples revealed significant alterations in several key biological processes associated with treatment response (**Figure 8**). The analysis demonstrated that immune response pathways showed the highest level of enrichment (enrichment score = 0.850, *P* = 0.001), followed by inflammation-related pathways (enrichment score = 0.720, *P* = 0.003), cell death regulation (enrichment score = 0.680, *P* = 0.005), metabolic processes (enrichment score = 0.610, *P* = 0.008), and signal transduction mechanisms (enrichment score = 0.580, *P* = 0.012). The prominent enrichment of immune and inflammatory pathways aligns with established understanding of tuberculosis pathogenesis, where host immune function plays a determining role in disease progression and treatment success. The concurrent enrichment across these five major pathway categories suggests that treatment response involves complex interplay between immunological, inflammatory, metabolic, and signaling networks, providing a molecular basis for the clinical observations and supporting the integration of transcriptomic data with clinical parameters for comprehensive treatment intervention benefit prediction.

## 4 Discussion

Our study demonstrates that comprehensive machine learning optimization can achieve strong predictive performance for identifying patients who would benefit from pharmacist intervention services. Through comprehensive optimization including data augmentation, extensive feature engineering, and sophisticated ensemble learning, we developed a clinical prediction model trained exclusively on clinical variables that achieved strong performance with 92.25% accuracy (95% CI: 89.1-95.4%) and 96.96% AUC (95% CI: 94.8-99.1%) in predicting pharmacist intervention group assignment, representing a 36.25 percentage point improvement, and a 64.3% relative improvement within our study, demonstrating the substantial impact of the optimization strategies employed. Separately, we analyzed publicly available transcriptomic data to provide supportive biological context, but these data were not used as model features. The clinical prediction model uses only clinical variables and provides a robust framework for identifying patients who would benefit from pharmacist intervention services.

Previous research has demonstrated the potential of machine learning in predicting tuberculosis pharmacist intervention status. Studies relying solely on clinical variables have reported accuracies ranging from 65% to 85% (Wiens and Shenoy, 2018; Hussain and Junejo, 2019), while those integrating transcriptomic features have shown improved discrimination, with AUC values between 0.75 and 0.90 (Singhania et al., 2018; Cai et al., 2020). Although ensemble methods have been employed to enhance model stability and performance (Wiens and Shenoy, 2018), few studies have implemented a comprehensive, multi-level ensemble architecture combined with systematic data augmentation and feature engineering as presented here.

The substantial performance improvement from our baseline models (56.4% accuracy, 59.9% AUC) to the ultimate ensemble (92.25% accuracy, 96.96% AUC) underscores the critical importance of the systematic, multi-faceted optimization strategy we employed. This 36.25 percentage point improvement in accuracy represents a 64.3% relative improvement within our study, demonstrating the substantial impact of the optimization strategies employed (Jian et al., 2021). The observed improvement trajectory demonstrates that each optimization stage—feature engineering, data augmentation, and ensemble learning—contributed incrementally and synergistically to the final performance. No single strategy was sufficient in isolation; rather, it was their integrated application that enabled the achievement of exceptional predictive accuracy. This finding aligns with emerging literature suggesting that maximal performance in clinical machine learning is often attained through complementary methodological enhancements rather than reliance on a single technical innovation (Rezende et al., 2022). The substantial gain attributable to data augmentation (+13.25 percentage points) particularly highlights the value of techniques that effectively expand limited clinical datasets, a common challenge in medical research (Shorten et al., 2021). Similarly, the significant contribution of ensemble learning (+12.67 percentage points) reinforces established principles regarding the robustness of model averaging and stacking approaches for improving generalization and predictive stability (Zhou, 2021).

The expansion of our dataset by 328.1% via multi-neighbor interpolation emerged as the most impactful single factor, contributing a 13.25-percentage-point accuracy gain. This strategy directly addressed the fundamental constraint of limited sample size (n=467), a ubiquitous challenge in clinical machine learning that often impedes the training of complex, high-capacity models (Shorten et al., 2021). The multi-neighbor interpolation approach (k=7) proved superior to conventional oversampling techniques by preserving the underlying multivariate data structure and maintaining the complex, non-linear relationships between clinical variables. Statistical validation, confirmed through Kolmogorov-Smirnov tests (*P* > 0.05 for all features), ensured the augmented data maintained the original statistical properties, a critical step for preventing the introduction of artifactual patterns that can lead to model overfitting and poor generalizability (Anaya-Isaza and Mera-Jiménez, 2022). This robust augmentation framework was instrumental in enabling the training of our complex ensemble architecture comprising 43 base models, a feat that would have been infeasible with the original dataset due to the high risk of overfitting. Furthermore, the strict maintenance of class balance (intervention-to-control ratio ~48:52) throughout the augmentation process guaranteed equitable representation of both patient groups, thereby mitigating potential model bias.

The generation of 122 optimized features from 10 original clinical variables constituted a pivotal advancement, enabling our model to capture complex, non-linear relationships and synergistic interactions that were not apparent in the raw data. Interaction features formed the largest category (36.9%), followed by statistical features (28.7%) and polynomial features (23.0%), reflecting a strategic emphasis on uncovering complex variable interdependencies. Critically, the feature importance analysis demonstrated that composite scores integrating multiple biomarkers—specifically, the inflammation score (importance: 0.245) and immune balance index (importance: 0.134)—were among the most powerful predictors, consistently outperforming individual clinical variables. This finding carries significant clinical implication, suggesting that holistic measures of pathological states provide superior prognostic value compared to isolated biomarker readings, a concept supported by recent research in complex disease phenotyping (Schmauch et al., 2020). The dominance of engineered features among the top predictors validates our comprehensive feature engineering strategy and underscores a fundamental principle in clinical machine learning: that capturing the complex, multi-factorial nature of diseases like tuberculosis requires moving beyond simple linear representations of clinical data to embrace higher-order feature interactions and integrated biological scores (Van Der Ploeg et al., 2014).

The implementation of a sophisticated multi-level ensemble architecture, which integrated 43 diverse base models through stacking, weighted averaging, and strategic blending, was instrumental in maximizing predictive performance by capitalizing on the complementary strengths of heterogeneous algorithms. This hierarchical framework operated across three distinct tiers: (1) Level 1 Stacking employing five meta-learners, (2) a Stacking Blend that combined these meta-learner predictions, and (3) an Ultimate Final Blend that optimally synthesized outputs from all ensemble components. This design ensured that final predictions benefited from both extensive model diversity—spanning different algorithmic families, hyperparameter configurations, and random initializations—and advanced combination methodologies. The ultimate ensemble demonstrated superior performance compared to any individual model or single ensemble technique, underscoring the value of such an integrated approach. Notably, the substantial improvement from the best individual model’s cross-validation AUC (0.9420) to the ultimate ensemble’s test set AUC (0.9696) highlights that the ensemble strategy provided significant gains beyond what was achievable through hyperparameter optimization alone. This observation aligns with established machine learning principles wherein ensemble methods enhance robustness and predictive accuracy by reducing variance and mitigating the risk of overfitting, particularly in complex clinical prediction tasks (Zhou, 2021; Rezende et al., 2022). The multi-level ensemble architecture, while complex, provides meaningful performance benefits. The 2.75 percentage point improvement from the best single model (89.5%) to the ultimate ensemble (92.25%) represents a 3.1% relative improvement. While this may appear modest, in high-performance clinical prediction models, such improvements are increasingly difficult to achieve and can have substantial clinical impact. The ensemble’s improved robustness and reduced overfitting risk further justify the added complexity. However, we acknowledge that for some clinical applications, computational efficiency may outweigh marginal performance gains, and the best single model (89.5% accuracy) provides a viable alternative.

The systematic hyperparameter optimization conducted via the Optuna framework with 250 trials per model was critical for identifying optimal configurations for the LightGBM, XGBoost, and CatBoost algorithms. This process employed 5-fold stratified cross-validation to ensure robust performance estimation and mitigate overfitting. The optimization comprehensively explored a wide parameter space, including learning rates (0.01-0.1), tree depths (5-20 for gradient boosting models), and various regularization parameters (L1 and L2), enabling the identification of configurations that maximized discriminatory power while preserving generalizability. This rigorous approach yielded a best model with a cross-validation AUC of 0.9420, indicating strong generalization potential and providing a reliable foundation for the subsequent ensemble construction. The effectiveness of this Bayesian optimization approach aligns with its growing application in tuning complex machine learning models for clinical prediction tasks, where it efficiently navigates high-dimensional hyperparameter spaces to discover high-performing configurations (Akiba et al., 2019).

The implementation of fine-grained threshold optimization across 201 candidate points within the [0.2, 0.8] range was essential for tailoring the model’s classification behavior to clinical priorities. To align model predictions with clinical priorities, we optimized the classification threshold to maximize a balanced performance profile. The selected threshold of 0.479 resulted in sensitivity of 92.0% and specificity of 92.5%, ensuring that both false-negative and false-positive errors were minimized—a crucial consideration for patient management (Steyerberg, 2019). The strategic weighting in the objective function reflects the clinical reality that overall predictive accuracy is often the primary concern, while still ensuring the model maintains strong discriminative capacity across the entire operating range. This approach to threshold calibration moves beyond conventional default settings and provides a methodology for aligning machine learning outputs with specific clinical utility requirements (Kuhn and Johnson, 2013).

The strong performance of our ultimate ensemble model, achieving 92.25% accuracy with balanced sensitivity (92.0%) and specificity (92.5%), underscores the critical importance of implementing comprehensive optimization strategies in clinical prediction tasks. This performance level not only represents a substantial improvement over our baseline models but also exceeds the predictive accuracy reported in most previous studies focused on TB pharmacist intervention benefit prediction. The model’s balanced performance across both patient groups, as evidenced by the low false positive (3.75%) and false negative (4.0%) rates in the confusion matrix, is particularly noteworthy and essential for equitable and effective clinical deployment. This characteristic ensures reliable performance regardless of a patient’s eventual outcome status, a crucial consideration for clinical decision-making where both types of classification errors carry significant implications for patient management and resource allocation (Steyerberg and Vergouwe, 2014). The demonstrated performance establishes a new benchmark for predictive modeling in tuberculosis care and highlights the transformative potential of systematically optimized machine learning approaches for complex clinical prediction challenges.

The feature importance analysis provides crucial insights into the biological mechanisms underpinning treatment response. The dominance of composite scores—specifically the inflammation score and immune balance index—along with ratio features such as the CD4^+^/CD8^+^ ratio and NLR among the top predictors, aligns well with the established understanding of tuberculosis pathogenesis, wherein host immune function and inflammatory regulation are central to treatment success (O’Garra et al., 2013). Furthermore, the prominence of interaction terms, particularly the age × CRP interaction, indicates that the relationships between clinical variables and intervention status are complex and context-dependent, involving synergistic effects that cannot be captured by examining individual biomarkers in isolation. These findings carry significant implications for clinical practice, as they emphasize the necessity of moving beyond univariate biomarker assessment toward integrated, multi-dimensional measures of immune status and inflammatory activity when evaluating treatment response and prognosis (Berry et al., 2010). This approach is consistent with the emerging paradigm of precision medicine in infectious diseases, which seeks to leverage complex, multi-modal data for improved patient stratification and management.

The identification of key biomarkers and their associated biological pathways provides valuable insights into the molecular mechanisms governing treatment response and reveals potential targets for future therapeutic strategies. Our pathway enrichment analysis demonstrated significant activation across multiple biological processes, with immune response pathways showing the strongest association (enrichment score = 0.850, p = 0.001), followed by inflammatory pathways (enrichment score = 0.720, p = 0.003). These findings align with the established understanding of TB pathogenesis, wherein host immune function and inflammatory regulation are recognized as critical determinants of disease progression and treatment success (Steyerberg and Vergouwe, 2014). Furthermore, the significant enrichment observed in cell death (enrichment score = 0.680, p = 0.005), metabolic (enrichment score = 0.610, p = 0.008), and signal transduction pathways (enrichment score = 0.580, p = 0.012) indicates that treatment response involves a complex interplay between multiple biological systems. This multi-pathway involvement underscores the necessity of clinical prediction model with supportive transcriptomic insights to fully comprehend treatment mechanisms, moving beyond single-pathway explanations toward a more holistic understanding of TB pharmacist intervention benefit (Allami and Yousif, 2023).

The identification of 150 significantly differentially expressed genes (75 up-regulated and 75 down-regulated) in the intervention group reveals a balanced transcriptomic response to pharmacist intervention, suggesting a broad modulatory effect spanning both pro-inflammatory and anti-inflammatory processes. This balanced distribution indicates that the intervention may facilitate a coordinated immunological recalibration rather than unidirectional suppression or activation. The integration of clinical parameters with transcriptomic profiles through our multi-modal analytical framework provides a more comprehensive understanding of treatment mechanisms than either data modality could offer independently, enabling the identification of biomarkers and pathways that remain obscured when examining clinical or genomic data in isolation (Kelly et al., 2019). This approach aligns with the growing recognition that complex clinical outcomes in tuberculosis are best understood through integrated analyses that capture both the phenotypic manifestations and their underlying molecular determinants (Singhania et al., 2018). The synergistic insights gained from this combined analysis not only enhance our understanding of the biological effects of pharmacist interventions but also establish a foundation for developing multi-modal biomarkers for treatment response prediction.

Several limitations of this study warrant consideration when interpreting our findings. First, the retrospective, single-center design introduces potential selection bias and may limit the generalizability of our results. Although we employed comprehensive optimization and robust internal validation strategies, including stratified cross-validation and independent test sets, our patient population from a single tertiary care center may not fully represent the broader TB patient spectrum across diverse healthcare settings (Steyerberg and Vergouwe, 2014). Second, while data augmentation substantially improved model performance, the original cohort size (n=467) remained modest for training complex ensemble models, and external validation in independent, multi-center cohorts is essential to verify generalizability (Anaya-Isaza and Mera-Jiménez, 2022). Third, the extensive feature engineering pipeline, while critical to performance, generated 122 features from 10 original variables, potentially affecting clinical interpretability and implementation feasibility. Although feature importance analysis identified key predictors, the complexity of engineered features and the multi-level ensemble architecture may challenge clinical adoption and real-time deployment (Kelly et al., 2019). Finally, as clinical practices and TB strains evolve over time, the temporal validity of our model requires ongoing evaluation, necessitating periodic recalibration with contemporary data to maintain predictive accuracy. Future studies should prioritize prospective, multi-center validation and explore simplified yet powerful modeling approaches to facilitate clinical translation.

To advance this field and enable clinical translation, several strategic research directions should be prioritized. First, rigorous prospective validation in independent, multi-center cohorts is essential to verify the model’s generalizability and real-world performance across diverse healthcare settings and patient demographics (Steyerberg and Vergouwe, 2014). Second, focused efforts on clinical implementation should address key challenges including electronic health record integration, user-friendly interface design, and computational optimization of the ensemble architecture to enable real-time decision support without sacrificing predictive accuracy (Kelly et al., 2019). Third, model interpretability requires significant enhancement through techniques such as SHAP (SHapley Additive exPlanations) and LIME (Local Interpretable Model-agnostic Explanations) to provide clinicians with transparent, biologically plausible explanations for predictions, thereby facilitating trust and adoption (Lundberg and Lee, 2017). Fourth, the biomarker candidates identified through our feature importance and pathway analyses warrant systematic biological validation to confirm their mechanistic roles in treatment response and assess their potential as therapeutic targets (Kelly et al., 2019). Finally, establishing a dynamic learning framework for continuous model recalibration will be crucial to maintain performance as treatment guidelines, patient populations, and microbial resistance patterns evolve over time (Davis et al., 2017). These coordinated efforts across validation, implementation, interpretation, and biological discovery will be essential for translating our high-performing predictive model into clinically impactful decision-support tools.

## 5 Conclusion

This study successfully developed and validated a clinical prediction model specifically designed to identify tuberculosis patients who would benefit from pharmacist intervention services. Through systematic and integrated optimization strategies—including data augmentation, extensive feature engineering, and multi-level ensemble learning—the final ensemble model, trained exclusively on clinical variables, achieved an accuracy of 92.25% and an AUC of 96.96% in predicting pharmacist intervention group assignment, demonstrating substantial performance improvement over baseline models. Feature importance analysis revealed that composite clinical indicators such as the inflammation score, immune balance index, and CD4/CD8 ratio were the most predictive, underscoring the value of multidimensional clinical assessment in accurately identifying patients who would benefit from intervention. Independent supportive transcriptomic analysis further provided biological contextualization for the model, revealing enrichments in immune and inflammatory pathways that aligned with the key clinical features, thereby enhancing the biological plausibility of the model’s findings. This study demonstrated that in-depth, synergistic optimization of existing machine learning workflows can significantly enhance model performance in clinical prediction tasks. The constructed framework not only exhibited excellent discriminative ability and balanced clinical metrics (sensitivity 92.0%, specificity 92.5%) but also provided a powerful data-driven tool for early and personalized patient stratification in healthcare resource allocation, showing clear potential for clinical translation.

## Data Availability

The data analyzed in this study is subject to the following licenses/restrictions: The datasets generated during the current study are not publicly available during the website review process, but are available from the corresponding author upon reasonable request. The full trial protocol is also available from the corresponding author upon request. Requests to access these datasets should be directed to lt881117@163.com.
